# Evolution of proteomes: fundamental signatures and global trends in amino acid compositions

**DOI:** 10.1186/1471-2164-7-307

**Published:** 2006-12-05

**Authors:** Fredj Tekaia, Edouard Yeramian

**Affiliations:** 1 Unité de Génétique Moléculaire des Levures (URA 2171 CNRS and UFR927 Univ. P.M. Curie), Institut Pasteur, 25, Rue du Dr Roux, 75724 Paris Cedex 15, France; 2 Unité de Bio-Informatique Structurale, URA CNRS 2185, Institut Pasteur, 25, Rue du Dr Roux, 75724 Paris Cedex 15, France

## Abstract

**Background:**

The evolutionary characterization of species and lifestyles at global levels is nowadays a subject of considerable interest, particularly with the availability of many complete genomes. Are there specific properties associated with lifestyles and phylogenies? What are the underlying evolutionary trends? One of the simplest analyses to address such questions concerns characterization of proteomes at the amino acids composition level.

**Results:**

In this work, amino acid compositions of a large set of 208 proteomes, with significant number of representatives from the three phylogenetic domains and different lifestyles are analyzed, resorting to an appropriate multidimensional method: Correspondence analysis. The analysis reveals striking discrimination between eukaryotes, prokaryotic mesophiles and hyperthemophiles-themophiles, following amino acid usage. In sharp contrast, no similar discrimination is observed for psychrophiles. The observed distributional properties are compared with various inferred chronologies for the recruitment of amino acids into the genetic code. Such comparisons reveal correlations between the observed segregations of species following amino acid usage, and the separation of amino acids following early or late recruitment.

**Conclusion:**

A simple description of proteomes according to amino acid compositions reveals striking signatures, with sharp segregations or on the contrary non-discriminations following phylogenies and lifestyles. The distribution of species, following amino acid usage, exhibits a discrimination between [high GC]-[high optimal growth temperatures] and [low GC]-[moderate temperatures] characteristics. This discrimination appears to coincide closely with the separation of amino acids following their inferred early or late recruitment into the genetic code. Taken together the various results provide a consistent picture for the evolution of proteomes, in terms of amino acid usage.

## Background

Mining the unprecedented wealth of information contained in complete genomes may help understand the evolutionary history of species. Available genomes from the three phylogenetic domains, covering a wide spectrum of lifestyles, provide through global comparative analyses, new opportunities to decipher genomic characteristics related to the adaptive evolution of organisms, notably for extreme conditions such as high [1] or low [2] temperatures. At the DNA level the simplest analyses concern the GC compositions and, similarly, at the proteomes level the simplest analyses concern the amino acid compositions. Yet, even such simple comparative descriptions can reveal important evolutionary properties for the genomes. For example, at local levels, significant variations in GC (or dinucleotide) compositions were associated with horizontal transfers or pathogenicity islands in bacteria (see for example [3]). At more global levels, GC compositions revealed complex isochore organisations in various eukaryotes (see for example [4]). For proteomes, compositional description is more elaborate, because of the number of amino acids as compared to the number of bases. Also, in comparison to the simple one-dimensional linearity associated with DNA, the analysis of compositional properties in proteomes makes it necessary to resort to appropriate multi-dimensional representations for the data. Such analyses have been performed from different perspectives, trying for example to identify signatures associated with different lifestyles. In such background, it was shown that amino acid usage is under the influence of GC content, reflecting adaptations to specific environmental conditions [5]. Also, analyses of amino acid compositions in a limited number of proteomes (as available at the time [1,6-8]) revealed a discrimination of hypethermophiles.

In this work, we consider an extensive analysis of amino acid usage in 208 proteomes, taking advantage of the recent significant number of available complete genomes with the notable increased representation for eukaryotes, psychrophiles and hyperthermophiles. The work here extends our previous study concerning 54 genomes [7]. Resorting to Correspondence analysis, we derive the distribution of species following amino acid usage, characterizing the associated fundamental discriminant signatures and the underlying evolutionary trends.

The analysis here fully confirms the previous observation for the discrimination between hyperthermophiles and mesophiles, with, in addition, a striking clearcut segregation of eukaryotes from all other species. In sharp contrast, it appears that psychrophilic lifestyle is not associated with specific profiles as the corresponding species are not distinguished from mesophilic prokaryotes, at the level of amino acid compositional analyses. We analyze statistically the segregated groups (eukaryotes, prokaryotic mesophiles and hyperthermophiles-thermophiles), highlighting the associated discriminant signatures. Finally we attempt to characterize the evolutionary trends underlying the observed segregations. We observe that the distribution of species, acccording to amino acid usage, can be associated essentially with a separation of amino acids following their early or late recruitment into the genetic code, as inferred in a series of recently published works [9,10]. This separation, from early to late, can be associated with a directionality from high GC contents and high optimal growth temperatures towards lower GC contents and moderate temperatures.

The evolutionary implications for the various observations, in terms of segregation and time directionality in relation to amino acid recruitment into the genetic code, are discussed. The results could also be relevant to practical grounds for protein comparison methods, with the perspective of refined amino acid substitution scores.

## Results

**Figure 1:**
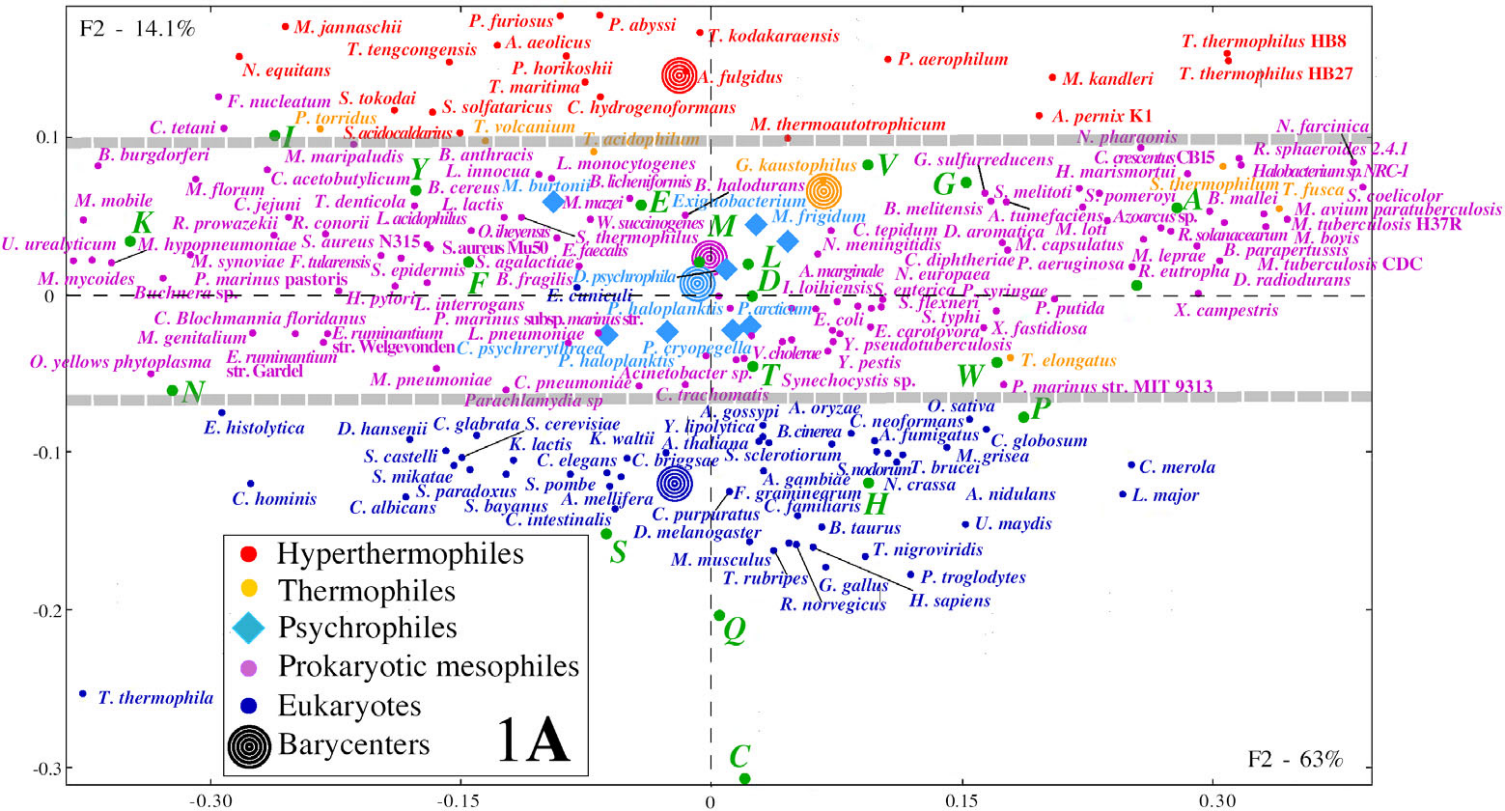
**Species distribution following amino-acid compositions**. Factorial plane representation, with Correspondence analysis, for the distribution of species following amino acid compositions. Species names are reported whenever possible (for full list see Additional file 1). Amino acids (one letter) are represented in green. Coding colours for hyperthemophiles, thermophiles, prokaryotic mesophiles, psychrophiles and eukaryotes are following legend in Figure 1A. Three main groups, corresponding to hyperthermophiles-thermophiles; prokaryotic mesophiles-psychrophiles and eukaryotes are roughly delimited by horizontal dashed grey lines. **A: Legend for colour coding symbols**. Species and barycenters symbols for the various groups that are reported in Figures 1, 2, 3 and 4 with the same colour coding conventions.

We used Correspondence analysis (see Methods) to compare amino acid compositions of 208 predicted proteomes with large representations of the three phylogenetic domains as well as various lifestyles (20 hyperthermophiles, 7 thermophiles, 8 psychrophiles and 173 mesophiles including 53 eukaryotes; detailed list is in Additional file 1). Figure 1 shows the resulting distribution of species and amino acids as projected on the first factorial plane, representing 77% of the total information in the original data table. We analyze first the distribution of species, in terms of global properties and discriminated groups. We then focus on more detailed statistical characterizations of the various groups, with their associated amino acid signatures. Finally we explore potential evolutionary trends associated with the various observations.

### Distribution of species and segregations

#### Global description

Confirming and refining the results of previous analyses [6,7] the global distribution of species is first following GC content, as corresponding to F1 factorial axis (contribution of order 63%), increasing from left to right (23% in *Mycoplasma mycoides *to 72.1% in *Streptomyces coelicolor*), and secondly following optimal growth temperatures, as corresponding to F2 factorial axis (contribution of order 14%), increasing upward from moderate to high temperatures. It is important to stress that GC content and optimal growth temperatures are not included in the set of analysed parameters, but correspond rather to observations underlying the distributions of species as obtained from their amino acid compositions.

#### Species segregation and discrimination following lifestyles and phylogenies

Based on phylogenetic and lifestyle classifications (as identified by colour codes in Figure 1 and Figure 1A), we observe a striking segregation for eukaryotes, prokaryotic mesophiles and hyperthermophiles, with sharply defined non-overlapping associated strips. With respect to this segregation, the only 'discrepancy' concerns the eukaryotic *E. cuniculi*, in the territory of mesophilic prokaryotes. The thermophilic species are essentially at the border between hyperthermophiles and prokaryotic mesophiles (with the exception of *T. elongatus*). In contrast with such clearcut segregation, we observe that psychrophiles are in the strip associated with prokaryotic mesophiles.

Concerning this general stratified structure in the distribution of species, we observe (Figure 1) that the barycenters of the various categories considered above (hyperthermophiles (HTH), thermophiles (TH), prokaryotic mesophiles (PMES), psychrophiles (PSYC) and eukaryotes (EUK)) are roughly aligned along the second factorial axis. This structure shows that species are rather homogeneously distributed within each category around the barycenters axis (with a mean value of about 40%) according to their GC content. With this respect, based on the 8 available species, it appears that the scattering of psychrophiles around the corresponding barycenter is of limited extent as compared to the other categories.

### Statistical characterization of segregated groups and associated signatures

#### Statistical characterization of segregated groups

For detailed characterization of the species distribution observed in Figure 1 we compared for the various groups (HTH, TH, PMES, PSYC and EUK) the mean amino acid compositions, along with pooled means associated with physico-chemical characteristics (polar, charged and hydrophobic). We used one way analysis of variance, followed by Newman-Keuls multiple comparison test for pairwise differences. For robustness and consistency reasons we choose a high probability limit of significance, set at the probability p < 0.001. Such comparisons revealed significant signatures (with steady variations of mean values, either increasing or decreasing) between the three following groups: a merged group associated with hyperthermophiles and thermophiles (HTH-TH); a merged group associated with prokaryotic mesophiles and psychrophiles (PMES-PSYC) and finally eukaryotes (EUK). The corresponding signatures and characteristic trends are detailed below.

#### Physico-chemical signatures

**Figure 2:**
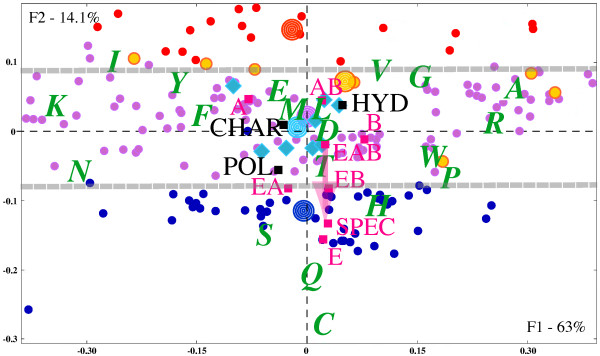
**Species distribution, physico-chemical signatures and proteins conservations**. Factorial plane representation and species colour coding are as in Figure 1 (species are only reported with their positions; see legend for colour coding symbols in Figure 1A). Amino acids (one letter) are in green. The three pools for polar (POL), charged (CHAR) and hydrophobic (HYD) amino acids are reported in black. The positions of the subsets corresponding to specific proteins (SPEC), as well as to proteins exclusively conserved in one (E, A and B) or in combinations of phylogenetic domains (EA, EB, AB and EAB) are reported in pink.

The pools of polar, charged and hydrophobic amino acids are represented on the factorial plane in Figure 2. The pools associated respectively with polar and the difference [polar – charged] amino-acids are characteristic of each one of the three segregated groups (HTH-TH, PMES-PSYC and EUK), all three mean value pairs being significantly different at p < 0.001. The abundance of the polar and [polar – charged] pools increase steadily from HTH-TH to PMES-PSYC, from HTH-TH to EUK and from PMES-PSYC to EUK (see Additional file 4). As for the hydrophobic pool, we observe a decrease from HTH-TH to EUK and from PMES-PSYC to EUK. The relative abundance in the hydrophobic pool thus appears as a characteristic signature for eukaryotes (EUK), since the corresponding mean value is significantly different from that of each of the two other groups (HTH-TH and PMES-PSYC; the mean values for these two groups being not significantly different at p < 0.001).

#### Amino acid signatures

**Figure 3:**
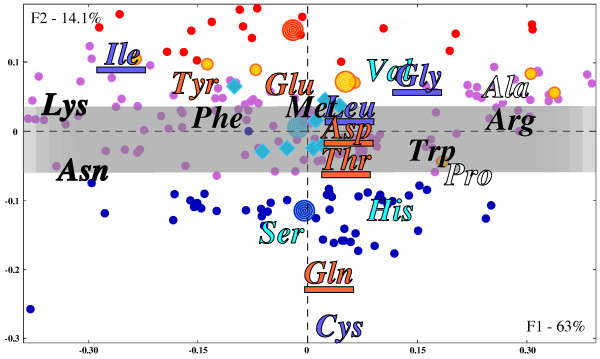
**Species distribution and characteristic amino-acid compositional signatures**. Factorial plane representation and species colour coding are as in Figure 1. Amino acids (three letters) are discriminated according to statistically significant mean compositional differences (at the probability level of p < 0.001, see text and Additional file 3) between the three segregated groups (hyperthermophiles-thermophiles, HTH-TH; prokaryotic mesophiles-psychrophiles, PMES-PSYC; eukaryotes, EUK): amino acids in pale blue (Val, His, Ser) are characteristic for each of the three groups; amino acids in orange (Tyr, Glu, Asp, Thr, Gln) are characteristic for HTH-TH; amino acids in dark blue (Gly, Ile, Leu, Cys) are characteristic for EUK; amino acids in black (Lys, Asn, Phe, Met, Trp, Arg) show no significant differences between the three groups (Ala and Pro show only partial significant differences: between EUK and PMES-PSYC); for a given class, underlined amino acids correspond to high values steadily decreasing in the three groups downward (HTH-TH to PMES-PSYC and to EUK) whereas non-underlined amino acids correspond to low values steadily increasing in the same direction.

Based on the discrimination of the three segregated classes HTH-TH, PMES-PSYC and EUK we classify (with the significance level at p < 0.001) amino acids following three groups (Figure 3 and Additional file 3):

a) Amino acids whose relative abundance is characteristic of each one of the three groups (with steady variation -either increase or decrease – from HTH-TH to PMES-PSYC and from PMES-PSYC to EUK): VAL (decrease), His and Ser (increase).

b) Amino acids characterizing HTH-TH or EUK: for EUK, Cys is high and Leu, Gly and Ile are low; whereas for HTH-TH, Tyr and Glu are high, Asp, Thr and Gln are low.

c) Amino acids with no discriminative characteristics (no significant differences between the three groups): all others (with the exception of Ala and Pro, with partial discriminative properties).

In summary, the characterizations above (at the probability significance level of p < 0.001) are represented in Figure 3 in correspondence with the segregation following the three main groups (HTH-TH, PMES-PSYC and EUK), with the non-discriminative amino acids essentially concentrated in a median horizontal strip in the factorial plane. The description would of course vary according to the threshold (for example, with a probability threshold of 0.05 Cys is found to increase from HTH-TH to PMES-PSYC). More detailed amino acid comparison results are reported in Additional file 2, file 3 and file 4.

### Overall trends and amino acid chronologies

We investigate overall trends which could underly the segregation of species, following amino acid territories as shown in Figure 1.

#### Protein conservation

For the three phylogenetic domains of life (Achaea (A), Bacteria (B) and Eukarya (E)), based on systematic comparisons of proteomes, the subsets of proteins conserved exclusively in one or in combinations of domains (E, A, B, EA, EB, AB and EAB) were determined, along with the subset of species specific proteins (SPEC, i.e. with no detectable similarities outside their own proteomes). The comparative data were from results in a recent study [11], concerning 100 species (amongst the 208 considered here). The amino acid compositions for the different subsets were determined and used as dummy observations (see methods) in the factorial analysis distribution shown in Figure 2. Following this analysis, the trend from the core set EAB (which can be associated with ancient proteins [12,7] to the specific set SPEC of proteins is essentially following the factorial axis F2, and pointing towards eukaryotic territory.

#### Amino acid chronologies

In this section we consider the distributions observed above in the light of inferred chronologies for amino acids recruitment into the genetic code, following models and data from Jordan et al. [9], Trifonov [10], Miller [13,14] and Cronin and Pizzarello[15].

**Figure 4:**
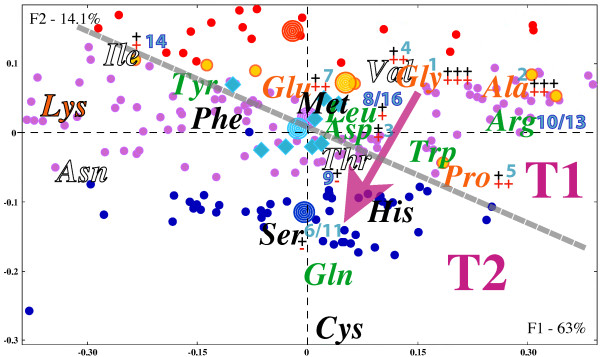
**Amino acids distribution and chronological models for the recruitment into the genetic code**. Factorial plane representation and species colour coding symbols are as in Figure 1. Dashed diagonal line in grey separates roughly the territory T1 ([high optimal growth temperature]-[high GC content]) from the territory T2 ([moderate optimal growth temperature]-[low GC content]), with arrow pointing from T1 to T2. Colour coding for amino-acids is following the model of Jordan et al. [9]: "strong losers" are orange (Glu, Gly, Ala, Pro); "weak loser" Lys is orange with a black outline; "strong gainers" are filled black (Cys, Ser, His, Phe, Met); "weak gainers" have black outlines (Asn, Ile, Thr, Val); all other amino acids are green. Amino acids found in Miller's experiments [14] or in the Murchison meteorite [15] are labelled with black, respectively red, crosses (the number of crosses, in each colour, follows the reported abundances for the corresponding amino acids; see Additional Table 3 in Jordan et al. [9]). Numbering of amino acids in blue is following the chronology compiled by Trifonov (Figure 1 in [10]; with possible different codon chronologies associated with a given amino acid, such as for Ser ranking 6 or 11 following the codon).

1) Model of Jordan et al.:

Following the model of Jordan et al. [9] amino acids are classified as "gainers" (either strong or weak) or "losers" (either strong or weak), with "gainers" corresponding to amino acids supposed to be recruited late into the genetic code. In this model the "strong gainers" are His, Ser and Cys (corresponding to the discriminant signatures for the three main segregated classes above) along with Phe and Met. Conversely, in this model, the "strong losers" (presumed to include the most ancient amino acids) are Pro, Ala, Glu and Gly. This separation between "strong losers" and "gainers" is recovered rather faithfully in our factorial analysis if we separate the factorial plane into two regions T1 and T2 (as represented in Figure 4) corresponding respectively to [high_temperature]-[high_GC] and [moderate_temperature]-[low_GC] characteristics, with a roughly defined border. With such a separation of the factorial plane we observe that the "strong gainers" His, Ser and Cys are in T2, with Phe and Met at the border. In addition, the "weak gainers" in this model (Asn, Thr and Ile; but not Val, in T1) also lie within the T2 space. Conversely, all "strong losers" lie within T1, while the "weak loser" Lys is situated in T2.

2) Model of Trifonov:

With the factorial plane separation above, we observe that the amino acids in T1 correspond largely to the first amino acids recruited into the genetic code according to the chronology suggested by Trifonov [10] (Gly, Ala, Asp, Val, Pro, Ser, Glu, Leu, Thr, Arg; see the order reported on the amino acids in Figure 4). In this list, the first discrepancy according to the separation in Figure 4 concerns the amino acid Ser in position 6 (with the 5 first amino acids in the list all situated in T1). It is nevertheless interesting to note that in the analysis of Trifonov it appears that Ser was also the first amino acid in the chronological classification for which two distinct positions were considered, following the associated codons (either UCX or AGY; with the associated ranks 6 and 11; see Figure 1 in [10]). As for Thr (ranked 9 in the chronology by Trifonov) its position in Figure 4 is at the border between T1 and T2. The two discrepancies between the chronology by Trifonov and the separation T1/T2 concern Ser and Thr and appear to correspond to contradictions between the models by Trifonov and by Jordan et al. (with Ser and Thr reported as "strong" and "weak gainers", respectively, in the model of Jordan et al. [9]).

3) Miller's experiments and data from the Murchison meteorite:

The ancient amino acids, as derived from Miller's experiments [13,14] and analysis of Murchison meteorite [15] (both informations being included in the criteria used for establishing the chronology in [10]), are essentially clustered in T1. It is striking to observe that the most abundant amino acids in these experiments [14,15] (Gly and Ala) are deep situated in T1, whereas those reported to be less abundant tend to cluster at the boundary between T1 and T2. It is also not surprising to observe that the possible "discrepancies" between the spark data (Miller's experiment) and the T1/T2 scheme concern again the amino acids Ser and Thr. However, interestingly, these amino acids do also correspond to the observed discrepancies between the spark data and the Murchinson meteorite data (Ser and Thr are not reported in the meteorite data; see representations in Figure 4) as well as to discrepancies between the two models above, as already mentioned. Finally, the overall decreasing gradient from T1 to T2 (Figure 4) in terms of ancient amino acid abundance is further enhanced with the chronology following the "yields of amino acids in imitated primordial conditions" as compiled by Trifonov [10] (criterion N3'; including 3 experimental conditions in addition to that of Miller's).

The various schemes above, relevant to the analysis of chronologies in correspondence with the recruitment of amino acids into the genetic code, let us suggest a time-directionality, the arrow from T1 to T2 in Figure 4. Overall, the direction of this arrow points in the same direction than the one associated with proteins conservation: from the most conserved ancestral common "core" of proteins to the set of species-specific proteins.

## Discussion

Based on amino acid compositions of proteomes, Correspondence analysis revealed a clearcut segregation of species following three lifestyle-phylogenetic classes: eukaryotes, prokaryotic mesophiles-psychrophiles and hyperthermophiles-thermophiles. Detailed statistical analyses confirmed the separation of the three classes, with associated signatures in terms of amino acids usage. Notably, the three classes are discriminated by the relative abundances in His, Ser and Val, and in the pools associated with polar and [polar – charged] amino acids. With respect to such signatures, it is interesting to note that the [polar – charged] criterion is not exclusive to the hyperthermophiles-thermophiles class [8], but is a distinctive feature of each one of the three classes.

The evidence for sharply defined non-overlapping territories for species based on amino acids usage raises many questions relevant to the evolution of lifestyles and to phylogenies: for example are the segregations following general trends and what underlies such observed segregations?

Concerning the overall trends, a separation of the factorial plane into two regions (T1: [high_temperature]-[high_GC] and T2: [moderate_temperature]-[low_GC], respectively) revealed striking correspondences between the distribution of the amino acids in the respective regions and various classifications relative to chronologies for the recruitment of amino acids into the genetic code. In such correspondences, through convergent criteria, it appears that amino acids found in T1 are essentially those supposed to be the earliest in the genetic code, and, conversely, that amino acids found in T2 are essentially those supposed to be most recent. An interesting feature in the convergent picture is the emergence of a possible basic consensus between the various considered schemes relative to amino acid chronologies (no matter possible limitations inherent to each one of them; with this respect see for example the recent discussion of the work of Jordan et al. [9] in [16-18]). In this direction, it may well be that the analysis here, based on completely independent criteria (observed amino acid compositions), could provide an efficient 'filtering' scheme for capturing genuine convergences between the various models. For example, following all models and data, it is clear that the core of inferred most ancient amino acids are in T1. Such a core could correspond to the list [Gly, Ala, Val, Pro, Glu], in which the only notable discrepancy is for Val following the Jordan et al. [9] model (reported as a "weak gainer"). The other way round, the amino acid Ser, which is next in the chronology by Trifonov, is situated deep in T2, in agreement with the model of Jordan et al. [9] (as a "strong gainer"). This contradictory assignment could be further resolved in favour of Ser not early in the list by noting that even though present in the spark experiment (in little amount) this amino acid was absent in the meteorite composition. And finally, in the Trifonov chronology itself, Ser was the first amino acid for which a dual rank was considered, following codon usage.

## Conclusion

In conclusion, an overall trend could be suggested for the observed segregation of species with amino acids usage, corresponding to an underlying time arrow from T1 to T2. This trend would be consistent with the following global evolutionary model: early steps of life were associated with high GCs and high temperatures [19], and, with the further recruitment of amino acids into the genetic code, the overall evolutionary trend was towards lower GCs, reduced temperatures and the appearance of new lifestyles, including mesophily and psychrophily, as well as the eukaryotic phylogenetic domain.

This study allowed to gain new insights into how amino acid usage has changed over evolutionary time. Such results could also help to better understand proteins evolution, notably in terms of physico-chemical and structural properties in adaptation to various conditions. With this respect, a discriminative feature associated with psychrophiles, despite their non-segregation, appears to be the limited extent of their spreading on the factorial plane around mean values. If further confirmed, this observation concerning the 8 available genomes, could reveal complex characterizations for psychrophilic proteins with constrained mutual dependences in amino acid compositions.

Finally, on application grounds, the results here could lead to enhanced amino acid substitutional models, taking into account amino acid frequencies reflecting the topology of species segregations as demonstrated here.

## Methods

The predicted proteomes for 208 species (Additional file 1) were mainly downloaded from the ncbi web server [20]. Lifestyles and optimal growth temperature are as reported on this server. The classification of species according to optimal growth temperatures was as follows: hyperthermophiles for temperatures higher than 60°C; thermophiles for temperatures between 60°C and 50°C; mesophiles for temperatures between 50°C and 15°C and psychrophiles for temperatures lower than 15°C.

The amino acid compositions of the 208 species were calculated, leading to a data table (208 rows versus 20 columns). The data table was analyzed using Correspondence analysis [21-23].

### Correspondence analysis

Correspondence analysis [21-23] is a powerful method for the multivariate exploration of large-scale data. This method has been applied in various research areas, including genomic analyses (for example [24-26]). For the extraction of relevant informations from the raw data, Correspondence analysis relies on the projection of high-dimensional informations on low-dimensional spaces. Such projections, into a plane, allow direct visual inspection of significant trends, which are often difficult to grasp in the high-dimensional spaces. The dimensions of the considered spaces are relevant to the number of variables and observations involved in the study (such as, here, the variables associated with the different amino-acids, and the observations associated with the different species). In this multivariate method -as applied to positive numerical data matrices – we can construct an orthogonal system called factorial axes, corresponding to the low-order projections on planes called factorial planes. An important virtue of this construction is that the characteristic properties of the observations and the variables are displayed simultaneously on the factorial planes. A transition formula allows the calculation of the coordinates of a given observation (respectively variable) as a function of the variables (respectively observation) coordinates. The method is called after the 'correspondence' between the analysis of observations and that of variables. In this analysis, each factorial axis represents a fraction of the whole information in the analysed table. The statistical significance of this fraction determines the relative confidence attached to the displayed observations and/or variables, on the corresponding axis. The orthogonality of the factorial axes permits the summation of their corresponding information fractions. For example, the fraction of total information included in the first factorial plane is obtained by summing the fractions corresponding to the first (F_1_) and to the second (F_2_) factorial axes. Positions on the factorial space are directly linked to the similarities between species, amino acids and relationships between these two sets. Species with similar global amino acid compositions are displayed close to each other. Species with high compositions in some amino acids are plotted in the same direction with regard to the origin. Such representation reveals associations between subsets of species and/or amino acids. Correspondence analysis also allows to consider dummy or 'illustrative variables' (respectively 'illustrative observations'), as additional variables (respectively observations) which do not contribute to the construction of the factorial space, but can be displayed on this factorial space. With such a representation it is possible to determine to which observations and variables the illustrative variables and observations are close to. It is worth to mention that in classification studies, Correspondence analysis can be used as preliminary step to represent the observations in an orthologonal system, so that euclidean distances can be calculated to construct clusters of observations (as for example in [11,25]).

#### Illustrative variables and observations

Following the Correspondence analysis method, we considered "illustrative variables" (respectively "illustrative observations"), as additional variables (respectively observations), which can be displayed on the factorial space. Accordingly the following positions on the factorial plane were determined: barycenters for hyperthermophiles (HTH), thermophiles (TH), psychrophiles (PSYC), prokaryotic mesophiles (PMES) and eukaryotes (EUK). The positions for polar (POL), charged (CHAR) and hydrophobic (HYD) amino acids were also determined.

Three illustrative variables have been considered: 'CHAR' for charged amino acids (Asp (D), Glu (E), Lys (K), Arg (R) and His (H)), 'POL' for polar/uncharged amino acids (Gly (G), Ser (S), Thr (T), Asn (N), Gln (Q), Tyr (Y) and Cys (C)) and 'HYD' for hydrophobic amino-acids (Leu (L), Met (M), Ile (I), Val (V), Trp (W), Pro (P), Ala (A) and Phe (F)). The amino-acid composition values attributed to the supplementary variables were obtained by summing the respective contributions of the corresponding amino acids, in the various species (for example, for the variable CHAR, the contributions of the amino acids Asp, Glu, Lys, Arg and His, are summed).

Six illustrative observations were considered:

Amino acid compositions in hyperthermophilic species (HTH), thermophiles (TH), psychrophiles (PSYC), prokaryotic mesophiles (PMES) and eukaryotes (EUK).

Eight other illustrative observations corresponding to amino acids compositions in subsets of proteins were determined from available 100 species comparisons (among the 208) as obtained in a recent work [11]:

1) proteins specific to each species (SPEC: proteins with no matches outside their own genomes),

2) proteins conserved exclusively in one domain of life [E: in eukaryal species, A: in archaeal species and B: in bacterial species],

3) proteins exclusively conserved in a combination of 2 domains [EA: eukaryal and archaeal, EB: eukaryal and bacterial and AB: archaeal and bacterial], or in the intersection of the three domains [EAB: eukaryal, archaeal and bacterial].

The final data table submitted to Correspondence analysis is composed of 222 lines versus 23 columns, including 14 illustrative observations and 3 illustrative variables.

### Statistical tests

One way analysis of variance was used to compare mean amino-acid compositions between the considered groups (HTH-TH, PMES-PSYC, EUK). When a significant difference was observed, Newman-Keuls (NK) multiple comparison test was performed to determine pairs with significant mean differences. For robustness and consistency we only considered in this work significant differences at the probability level of p < 0.001. Detailed results are reported in Additional file 2, file 3 and file 4.

## Abbreviations

A: represents amino acid composition of a set of proteins exclusively conserved in archaeal species.

B: amino acid composition of a set of proteins exclusively conserved in bacterial species.

AB: amino acid composition of a set of proteins exclusively conserved in a combination of archaeal and bacterial species;

CHAR/char: charged amino acids;

E: amino acid composition of a set of proteins exclusively conserved in Eukaryal species;

EA: amino acid composition of a set of proteins exclusively conserved in a combination of eukaryal and archaeal species;

EB: amino acid composition of a set of proteins exclusively conserved in a combination of eukaryal and bacterial species;

EAB: amino acid composition of a set of proteins exclusively conserved in a combination of eukaryal, archaeal and bacterial species;

EUK: amino acid composition of the eukaryotes considered in this analysis;

F1: first factorial axis;

F2: second factorial axis;

HTH: amino acid composition of the hyperthermophiles considered in this analysis;

HYD/hyd: hydrophobic amino acids;

NK: Newman-Keuls multiple comparison test;

POL/pol: polar amino acids;

PMES: amino acid composition of the prokaryotic mesophiles considered in this analysis;

PSYC: amino acid composition of psychrophiles considered in this analysis;

TH: amino acid composition of the thermophiles considered in this analysis;

SPEC: amino acid composition of a set of proteins exclusively conserved in their own proteome.

## Authors' contributions

FT conceived the study and performed the data analyses. FT and EY participated equally in the design of the analyses, the interpretation of the results and in the writing of the manuscript. Both authors read and approved the final manuscript.

## Supplementary Material

Additional file 1 **List of species considered in the analysis**. The table includes : column 1: the phylogenetic domain of the species (Dom) with the following abbreviations: E (Eukaryotes), A (Archaea), B (Bacteria); column 2: species code; column 3: genomic GC contents, whenever available (GC%); column 4: optimal growth temperature, whenever available (OGT); column 5: total number of predicted proteins (Prot); column 6: species identification. blue stars correspond to psychrophiles; orange stars to thermophiles and red stars to hyperthermophiles. Click here for file

Additional file 2 **Average proportions of amino-acids in each of the considered groups (HTH-TH, PMES-PSYC, EUK) and their statistical comparisons**. Average amino-acid composition in each of the three groups (Hyperthermophiles-thermophiles (HTH-TH), Prokaryotic mesophiles-psychrophiles (PMES-PSYC), Eukaryotes (EUK)) and their comparisons using one-way analysis of variance followed by Newman-Keuls (NK) multiple comparison test for pairwise differences. The table shows for each amino-acid its mean value and the corresponding standard deviation in each group, followed by the degree of significant difference if any between each pair of groups (NK: ***: p < 0.001; **: p < 0.01; *: p < 0.05; ns:non-significant. The symbols "+", "-" identify respectively average increases and decreases). For robustness and consistency only significant differences at the probability level of p < 0.001 are considered. Click here for file

Additional file 3 **Mean compositions for each amino acid in each of the three groups: Hyperthermophiles-thermophiles (red), Prokaryotic mesophiles-psychrophiles (light blue) and Eukaryotes (blue)**. Mean values for each amino acid in each of the three groups: Hyperthermophiles-thermophiles (red), Prokaryotic mesophiles-psychrophiles (light blue) and Eukaryotes (blue). Symbol * is associated with significant Newman-Keuls multiple comparison tests at the probability p < 0.001 (see Methods). Amino acids underlined with black, red and blue are characteristic respectively, of the three groups (Hyperthermophiles-thermophiles; Prokaryotic mesophiles-psychrophiles and Eukaryotes), of Hyperthermophiles only and of Eukaryotes only. Click here for file

Additional file 4 **Mean amino acid compositions according to physico-chemical properties**. This figure shows mean values for hyd (hydrophobics), pol (polar), pol-char (pol – char) and char (charged) amino acids in each of the three groups: Hyperthermophiles-thermophiles; Prokaryotic mesophiles-psychrophiles and Eukaryotes. Colours are as in Additional file 3. Symbol * is associated with significant Newman-Keuls multiple comparison tests at p < 0.001 (see Methods). pol and [pol-char] are specific for each of the three groups. Click here for file
