# Supplementary material for: Evolution of proteomes: fundamental signatures and global trends in amino acid compositions
Source: BMC Genomics. 2006 Dec 5;7:307. doi: 10.1186/1471-2164-7-307 (PMC1764020; doi:10.1186/1471-2164-7-307)
Supplement: Additional file 1 — List of species considered in the analysis. The table includes : column 1: the phylogenetic domain of the species (Dom) with the following abbreviations: E (Eukaryotes), A (Archaea), B (Bacteria); column 2: species code; column 3: genomic GC contents, whenever available (GC%); column 4: optimal growth temperature, whenever available (OGT); column 5: total number of predicted proteins (Prot); column 6: species identification. blue stars correspond to psychrophiles; orange stars to thermophiles and red stars to hyperthermophiles. [file 1471-2164-7-307-S1.doc]

| Dom | Code | GC% | OGT | Prot | Organism |
| --- | --- | --- | --- | --- | --- |
| E | SC | 38.3 |  | 5829 | *Saccharomyces cerevisiae* |
| E | SP | 36.0 |  | 4962 | *Schizosaccharomyces pombe* |
| E | NCU |  |  | 10082 | *Neurospora crassa* (wit 08-07-03) |
| E | CA |  |  | 6165 | *Candida albicans* |
| E | MGR |  |  | 11109 | *Magnaporthe Grisea* (wit 08-10-03) |
| E | FG |  |  | 11640 | *Fusarium Graminearum* (wit 08-10-03) |
| E | AN | 50.0 |  | 9541 | *Aspergillus nidulans* (wit 08-10-03) |
| E | ECUN | 47 |  | 1996 | *Encephalitozoon cuniculi* |
| E | CE | 35.4 |  | 20844 | *Caenorhabditis elegans* |
| E | CBG | 37.4 |  | 25111 | *Caenorhabditis briggsae* (ensembl) |
| E | DM | 41.1 |  | 17878 | *Drosophila melanogaster* |
| E | AG | 35.2 |  | 16112 | *Anopheles gambiae* |
| E | ATH | 34.9 |  | 22671 | *Arabidopsis thalina* |
| E | CMER |  |  | 4772 | *Cyanidioschyzon merolae* (http://merolae.bio.s.u-tokyo.ac.jp) |
| E | HS | 41.0 |  | 27625 | *Homo sapiens* (ncbi version May 2002) |
| E | MUS | 42.0 |  | 28097 | *Mus musculus* (ensembl july 11 2002 version) |
| E | RN |  |  | 21205 | *Rattus norvegicus* (ncbi october 21 2003) |
| E | FR |  |  | 33609 | *Takifugu rubripes* |
| E | CI |  |  | 15851 | *Ciona intestinalis* |
| E | AFUM | 49.9 |  | 9746 | *Aspergilus fumigatus* |
| E | AORYZ | 48.0 |  | 14063 | *Aspergilus oryzae* |
| E | CRNE |  |  | 6594 | *Cryptococcus neoformans* var. neoformans JEC21 |
| E | AGOS | 52.0 |  | 4718 | *Ashbya gossypii* (*Eremothecium gossypii*) |
| E | CAGL | 38.8 |  | 5275 | *Candida glabrata* |
| E | YALI | 49.0 |  | 6695 | *Yarrowia lipolytica* |
| E | DEHA | 36.3 |  | 6889 | *Debaryomyces hansenii* |
| E | KLLA | 38.7 |  | 5334 | *Kluyveromyces Lactis* |
| E | KLWA |  |  | 5214 | *Kluyveromyces waltii* |
| E | SABA |  |  | 9424 | *Saccharomyces bayanus* (fungal_genomes MIT) |
| E | SACA |  |  | 4677 | *Saccharomyces castellii* (fungal_genomes MIT) |
| E | SAMI |  |  | 9057 | *Saccharomyces mikatae* (fungal_genomes MIT) |
| E | SAPA |  |  | 8955 | *Saccharomyces paradoxus* (fungal_genomes MIT) |
| E | TRBR | 46.4 |  | 8758 | *Trypanosoma brucei* (Protists, tigr) |
| E | TETH |  |  | 27424 | *Tetrahymena thermophila* (Protists,tigr) |
| E | ENHI |  |  | 9772 | *Entamoeba histolytica* (Protists, tigr) |
| E | CRHO |  |  | 3934 | *Cryptosporidium hominis* |
| E | TENI |  |  | 27918 | *Tetraodon nigroviridis* (Fish, genoscope) |
| E | ORSJ |  |  | 32290 | *Orysa Sativa-Japonica* (Rice, ncbi) |
| E | PATR |  |  | 21737 | *Pan troglodytes* (Chimp, ncbi) (animal) |
| E | BOTA |  |  | 35907 | *Bos taurus* (ncbi) (animal) |
| E | GAGA |  |  | 18031 | *Gallus gallus* (ncbi) (animal) |
| E | APME |  |  | 6298 | *Apis mellifera* DH4 (ncbi) (Insect) |
| E | CAFA |  |  | 33651 | *Canis familiaris* (ncbi) (animal) |
| E | STPU |  |  | 20989 | *Strongylocentrotus purpuratus* (ncbi) (animal) |
| E | STNO |  |  | 16597 | *Stagonospora nodorum* (broad Institut) |
| E | CHGL |  |  | 11124 | *Chaetomium globosum* (Broad Institut) |
| E | SCSC |  |  | 14522 | *Sclerotinia sclerotiorum* (Broad Institut) |
| E | USMA |  |  | 6522 | *Ustilago maydis* (Broad Institut) |
| E | BOCI |  |  | 16448 | *Botrytis cinerea* (Broad Institut) |
| E | LEMA | 59.7 |  | 8216 | *Leishmania major* |
| E | DOG | 41.0 |  | 33651 | *Dog* |
| A | H | 67 | 42 | 2058 | *Halobacterium* sp. NRC-1 |
| A | MA | 42 | 35-40 | 4528 | *Methanosarcina acetivorans* C2A |
| A | MMA | 41 | 30-40 | 3371 | *Methanosarcina mazei* strain Goe1 |
| A | HAMA | 61.1 | 40-50 | 3131 | *Haloarcula marismortui* ATCC 43049 (ncbi) |
| A | MEMA | 33 | 35-40 | 1722 | *Methanococcus maripaludis* S2 |
| A | NAPH | 63 |  | 2661 | *Natronomonas pharaonis* |
| B | HI | 38 | 35-37 | 1713 | *Haemophilus influenzae* |
| B | MG | 31 | 37 | 479 | *Mycoplasma genitalium* |
| B | MP | 40 | 37 | 677 | *Mycoplasma pneumoniae* |
| B | Ssp | 47 |  | 3168 | *Synechocystis* sp. |
| B | EC | 50 | 37 | 4290 | *Escherichia coli* |
| B | HP | 38 | 37 | 1577 | *Helicobacter pylori* |
| B | BS | 43 | 25-35 | 4100 | *Bacillus subtilis* |
| B | BB | 28 |  | 1639 | *Borrelia burgdorferi* |
| B | MT | 65 | 37 | 3996 | *Mycobacterium tuberculosis* H37R |
| B | MTC | 65 | 37 | 4203 | *Mycobacterium tuberculosis* CDC 1551 (TIGR) |
| B | TP | 52 |  | 1031 | *Treponema pallidum* |
| B | CT | 41.3 |  | 877 | *Chlamydia trachomatis* |
| B | RP | 29 |  | 837 | *Rickettsia prowazekii* |
| B | CJ | 30 |  | 1634 | *Campylobacter jejuni* |
| B | CP | 40 | 37 | 1052 | *Chlamydophila pneumoniae* |
| B | DR | 66.6 | 30-37 | 3117 | *Deinococcus radiodurans* |
| B | NM | 51 | 35-37 | 2081 | *Neisseria meningitidis* |
| B | XF | 52 | 26-28 | 2830 | *Xylella fastidiosa* |
| B | PAE | 66 | 25-30 | 5570 | *Pseudomonas aeruginosa* |
| B | VC | 47 | 20-30 | 3837 | *Vibrio cholerae* |
| B | BH | 43 |  | 4066 | *Bacillus halodurans* |
| B | B | 26 |  | 575 | *Buchnera* sp. |
| B | ML | 57 | 37 | 1604 | *Mycobacterium leprae* |
| B | YP | 47 | 28-30 | 3895 | *Yersinia pestis* |
| B | STY | 52 | 37 | 4395 | *Salmonella Typhi* |
| B | SAN315 | 32 | 30-37 | 2594 | *Staphylococcus aureus* N315 |
| B | SAMU50 | 32 | 30-37 | 2714 | *Staphylococcus aureus* Mu50 |
| B | LMO | 37 | 30-37 | 2846 | *Listeria monocytogenes* EGD-e |
| B | LIN | 37 | 30-37 | 2968 | *Listeria innocua* |
| B | SPY | 38 | 30-35 | 1696 | *Streptococcus pyogenes* M1 |
| B | AGRT | 59 | 25-28 | 5299 | *Agrobacterium tumefaciens* |
| B | MM | 62 |  | 7275 | *Mesorhizobium loti* |
| B | SM | 62 | 25-30 | 6205 | *Sinorhizobium meliloti* |
| B | SCO | 72 | 25-35 | 7810 | *Streptomyces coelicolor* |
| B | MB | 65 | 37 | 3953 | *Mycobacterium Bovis* |
| B | SHFL | 50 | 37 | 4068 | *Shigella flexneri* |
| B | UU | 25 |  | 614 | *Ureaplasma urealyticum* |
| B | LL | 35 | 40 | 2321 | *Lactococcus lactis* subsp. lactis |
| B | CCR | 67 | 35 | 3737 | *Caulobacter crescentus* CB15 |
| B | RCO | 32 |  | 1374 | *Rickettsia conorii* Malish 7 |
| B | NOS | 41 |  | 5366 | *Nostoc* sp. |
| B | BFL | 27 |  | 583 | *Candidatus Blochmannia floridanus* |
| B | PRO | 36 |  | 1882 | *Prochlorococcus marinus* subsp. *marinus* str. CCMP1375 |
| B | PMT | 50 |  | 2265 | *Prochlorococcus marinus* str. MIT 9313 |
| B | PMM | 30 |  | 1712 | *Prochlorococcus marinus* subsp. pastoris str. CCMP1378 |
| B | WS | 48 |  | 2044 | *Wolinella succinogenes* |
| B | PL | 42 |  | 4683 | *Photorhabdus luminescens* subsp. *laumondii* TTO1 |
| B | VVYJ | 46 | 20-30 | 5028 | *Vibrio vulnificus* YJ016 |
| B | VPR | 45 | 20-30 | 4832 | *Vibrio parahaemolyticus* RIMD 2210633 |
| B | STTHC | 39 | 45 | 1915 | *Streptococcus thermophilus* CNRZ1066 (ncbi) |
| B | STTHL | 39 | 45 | 1889 | *Streptococcus thermophilus* LMG 18311 (ncbi) |
| B | BUMA | 68 |  | 2996 | *Burkholderia mallei* ATCC 23344 |
| B | BRME | 57 | 37 | 2059 | *Brucella melitensis* 16M |
| B | BOPA | 68 | 35-37 | 4185 | *Bordetella parapertussis* 12822 |
| B | AZOA | 65 | 26 | 4133 | *Azoarcus* sp. EbN1 |
| B | YEPE | 47 | 28-30 | 3895 | *Yersinia pestis biovar Medievalis* str. 91001 |
| B | YEPS | 47 | 28-30 | 3901 | *Yersinia pseudotuberculosis* IP 32953 |
| B | NOFA | 70 | 37 | 5683 | *Nocardia farcinica* IFM 10152 |
| B | MYSY | 28 | 37 | 672 | *Mycoplasma synoviae* 53 |
| B | MYCM | 23 | 37 | 1016 | *Mycoplasma mycoides* subsp. *mycoides* SC str. PG1 |
| B | MYMO | 24 | 20 | 633 | *Mycoplasma mobile* 163K |
| B | MYHY | 28.5 | 37 | 665 | *Mycoplasma hyopneumoniae* |
| B | MEFL | 27 | 20-40 | 682 | *Mesoplasma florum* L1 |
| B | FUNU | 27 | 37 | 2067 | *Fusobacterium nucleatum* subsp. *nucleatum* ATCC 25586 |
| B | EHRU | 27 |  | 950 | *Ehrlichia ruminantium* str. *Gardel* |
| B | EHRW | 27 |  | 888 | *Ehrlichia ruminantium* str. *Welgevonden* |
| B | BACE | 35.4 | 25-35 | 5134 | *Bacillus cereus* E33L |
| B | BAAN | 35 |  | 5287 | *Bacillus anthracis* str. *Sterne* |
| B | BAAA | 35 |  | 5309 | *Bacillus anthracis* str. 'Ames Ancestor' |
| B | ACIN | 40 | 37 | 3325 | *Acinetobacter sp*. ADP1 |
| B | RAEU | 64 | 30 | 2407 | *Ralstonia eutropha* JMP134 |
| B | RHSP | 69 | 25-35 | 3022 | *Rhodobacter sphaeroides* 2.4.1 |
| B | SIPO | 64 |  | 3810 | *Silicibacter pomeroyi* DSS-3 |
| B | STAG | 35 | 37 | 2094 | *Streptococcus agalactiae* NEM316 |
| B | BALI | 46.2 |  | 4152 | *Bacillus licheniformis* ATCC 14580 |
| B | BAFR | 43 | 37 | 4578 | *Bacteroides fragilis* YCH46 |
| B | BDBA | 50 | 28-30 | 3587 | *Bdellovibrio bacteriovorus* HD100 |
| B | BAHE | 38 | 37 | 1488 | *Bartonella henselae* str. Houston-1 |
| B | CODI | 53 | 37 | 2272 | *Corynebacterium diphtheriae* |
| B | COBU | 42 | 37 | 2016 | *Coxiella burnetii* |
| B | DEAR | 59 |  | 4171 | *Dechloromonas aromatica* RCB |
| B | ENFA | 37 | 37 | 3113 | *Enterococcus faecalis* V583 |
| B | FRTU | 32.3 |  | 1603 | *Francisella tularensis tularensis* |
| B | GESU | 60 | 30 | 3446 | *Geobacter sulfurreducens* PCA |
| B | HADU | 38 | 35-37 | 1717 | *Haemophilus ducreyi* 35000HP |
| B | IDLO | 47 | 4-46 | 2628 | *Idiomarina loihiensis* L2TR |
| B | LEIN | 35 | 28-30 | 3394 | *Leptospira interrogans serovar Copenhageni* |
| B | MECA | 63 | 45 | 2960 | *Methylococcus capsulatus Bath* |
| B | MYAV | 69 | 37 | 4350 | *Mycobacterium avium paratuberculosis* |
| B | NIEU | 50 |  | 2461 | *Nitrosomonas europaea* |
| B | OCIH | 35 | 30 | 3500 | *Oceanobacillus iheyensis* |
| B | PARA | 35 | 30 | 2031 | *Parachlamydia* sp UWE25 |
| B | PSPU | 61 |  | 5350 | *Pseudomonas putida* KT2440 |
| B | PSSY | 55.6 |  | 5470 | *Pseudomonas syringae* pv. *phaseolicola* 1448A |
| B | RASO | 69 |  | 3440 | *Ralstonia solanacearum* |
| B | SAEN | 52 | 37 | 4093 | *Salmonella enterica* subsp. enterica serovar Paratyphi A str. ATCC 9150 |
| B | SHON | 45 |  | 4324 | *Shewanella oneidensis* MR-1 |
| B | STEP | 32 | 30-37 | 2494 | *Staphylococcus epidermidis* RP62A |
| B | STPN | 39 | 30-35 | 2094 | *Streptococcus pneumoniae* TIGR4 |
| B | TRDE | 37 | 30-42 | 2767 | *Treponema denticola* ATCC 35405 |
| B | TRWH | 46 | 37 | 783 | *Tropheryma whipplei* TW08/27 |
| B | VIFI | 38.4 |  | 2575 | *Vibrio fischeri* ES114 |
| B | XACA | 64 | 25-30 | 4273 | *Xanthomonas campestris* pv. *campestris* str. 8004 |
| B | YPES | 47 | 28-30 | 3885 | *Yersinia pestis* CO92 |
| B | LPP | 38.3 |  | 3082 | *Legionella pneumoniae Paris* |
| B | ANVA | 41 |  | 5039 | *Anabaena variabilis* ATCC 29413 |
| B | ANMA | 49 |  | 949 | *Anaplasma marginale* str. *St. Maries* |
| B | CLAC | 30 | 10-65 | 3672 | *Clostridium acetobutylicum* ATCC 824 |
| B | CLTE | 28 | 37 | 2373 | *Clostridium tetani* E88 |
| B | ERCA | 50 | 27-30 | 4472 | *Erwinia carotovora* subsp. *atroseptica* SCRI1043 |
| B | LAAC | 34 | 25-35 | 1864 | *Lactobacillus acidophilus* NCFM |
| B | ONYE | 27 |  | 754 | *Onion yellows phytoplasma* OY-M |
| B | PAMU | 40 | 37 | 2015 | *Pasteurella multocida* subsp. *multocida* str. Pm70 |
| B | CHTE | 56 | 48 | 2252 | *Chlorobium tepidum* TLS |
| A | MFR* |  | 15 | 1815 | *Methanogenium frigidum* |
| A | MBUR* | 40 | 23.4 | 2676 | *Methanococcoides burtonii* |
| B | DEPS* | 46 | 7 | 3116 | *Desulfotalea psychrophila* LSv54 (ncbi) |
| B | PSCR* | 42 | -10 | 2485 | *Psychrobacter cryopegella* (ncbi) |
| B | EXIG* | 47 |  | 2978 | *Exiguobacterium* (ncbi) |
| B | CPS* | 38 | 8 | 4910 | *Colwellia psychrerythraea* 34H (ncbi) |
| B | PSAR* | 42 | -2-10 | 2120 | *Psychrobacter arcticum* 273-4 (ncbi)(-2 to –10°C) |
| B | PSHA* | 40.1 | <10 | 3486 | *Pseudoalteromonas haloplanktis* TAC125 |
| A | PFU* | 40 | 100 | 2208 | *Pyrococcus furiosus* |
| A | PYAE* | 51 | 100 | 2605 | *Pyrobaculum aerophilum* |
| A | PH* | 41 | 98 | 2061 | *Pyrococcus horikoshii* OT3 |
| A | PA* | 44 | 103 | 1765 | *Pyrococcus abyssi* |
| A | APEM* | 56 | 90-95 | 1865 | *Aeropyrum pernix* K1 (ncbi) |
| A | MJ* | 31 | 85 | 1773 | *Methanococcus jannaschii* |
| A | AF* | 48 | 83 | 2409 | *Archaeoglobus fulgidus* |
| A | SSP2* | 35 | 85 | 2977 | *Sulfolobus solfataricus* P2 |
| A | STO* | 32 | 80 | 2826 | *Sulfolobus tokodaii* |
| A | MTH* | 49 | 65-70 | 1871 | *Methanobacterium thermoautotrophicum* |
| A | MK* | 61 | 98 | 1687 | *Methanopyrus kandleri* AV19 |
| A | NEK* | 31 | 90 | 563 | *Nanoarchaeum equitans* Kin4-M |
| A | THKO* | 51 | 85 | 2306 | *Thermococcus kodakaraensis* KOD1 (ncbi) |
| B | AE* | 43 | 96 | 1522 | *Aquifex aeolicus* |
| B | TM* | 46 | 80 | 1849 | *Thermotoga maritima* |
| B | TTE* | 37 | 75 | 2588 | *Thermoanaerobacter tengcongensis* strain MB4T |
| B | THTH* | 69 | 85 | 1982 | *Thermus-thermophilus* HB27 (ncbi) |
| B | THTH8* | 69 | 85 | 1973 | *Thermus-thermophilus* HB8 (ncbi) |
| A | SUAC* | 36 | 70-75 | 2223 | *Sulfolobus acidocaldarius* DSM 639 |
| B | CAHY* | 42 | 78 | 2620 | *Carboxydothermus hydrogenoformans Z-2901* |
| A | PITO* | 35 | 60 | 1535 | *Picrophilus-torridus* (ncbi) |
| A | TV* | 39 | 60 | 1526 | *Thermoplasma volcanium* |
| A | TA* | 45 | 59 | 1478 | *Thermoplasma acidophilum* |
| B | GEKA* | 52 | 60 | 3498 | *Geobacillus kaustophilus* HTA426 |
| B | SYTH* | 68 | 60 | 3337 | *Symbiobacterium thermophilum* IAM 14863 (ncbi) |
| B | TSE* | 53 | 55 | 2475 | *Thermosynechococcus elongatus* BP-1 |
| B | THFU* | 67 | 50-55 | 3110 | *Thermobifida fusca* YX |
