# Supplementary material for: Evolution of proteomes: fundamental signatures and global trends in amino acid compositions
Source: BMC Genomics. 2006 Dec 5;7:307. doi: 10.1186/1471-2164-7-307 (PMC1764020; doi:10.1186/1471-2164-7-307)
Supplement: Additional file 2 — Average proportions of amino-acids in each of the considered groups (HTH-TH, PMES-PSYC, EUK) and their statistical comparisons. Average amino-acid composition in each of the three groups (Hyperthermophiles-thermophiles (HTH-TH), Prokaryotic mesophiles-psychrophiles (PMES-PSYC), Eukaryotes (EUK)) and their comparisons using one-way analysis of variance followed by Newman-Keuls (NK) multiple comparison test for pairwise differences. The table shows for each amino-acid its mean value and the corresponding standard deviation in each group, followed by the degree of significant difference if any between each pair of groups (NK: ***: p < 0.001; **: p < 0.01; *: p < 0.05; ns:non-significant. The symbols "+", "-" identify respectively average increases and decreases). For robustness and consistency only significant differences at the probability level of p < 0.001 are considered. [file 1471-2164-7-307-S2.doc]

| **aa** | **HTH_TH^1^** | **PMES_PSYC^2^** | **EUK^3^** | **1 vs 2** | **1 vs 3** | **2 vs 3** |
| --- | --- | --- | --- | --- | --- | --- |
| **values** | **27** | **128** | **53** |  |  |  |
| **V (Val)** | **7.8** (1.0) | **6.9** (0.9) | **6.0** (0.7) | ***** -** | ***** -** | ***** -** |
| **hyd** | **45.4** (2.0) | **44.4** (2.1) | **40.2** (2.1) | *** -** | ***** -** | ***** -** |
| **Y (Tyr)** | **3.8** (0.9) | **3.2** (0.6) | **3.1** (0.7) | ***** -** | ***** -** | **ns** |
| **E (Glu)** | **7.7** (1.2) | **6.3** (0.9) | **6.6** (0.5) | ***** -** | ***** -** | *** +** |
| **G (Gly)** | **7.4** (1.0) | **7.0** (1.2) | **6.0** (1.1) | **ns** | ***** -** | ***** -** |
| **I (Ile)** | **7.4** (2.3) | **6.9** (1.9) | **5.6** (1.5) | **ns** | ***** -** | ***** -** |
| **L (Leu)** | **10.4** (1.4) | **10.2** (0.8) | **9.3** (0.6) | **ns** | ***** -** | ***** -** |
| **A (Ala)** | 7.8 (2.2) | **8.6** (2.6) | **6.9** (2.0) | **ns** | **ns** | ***** -** |
| **H (His)** | **1.7** (0.3) | **2.1** (0.3) | **2.4** (0.3) | ***** +** | ***** +** | ***** +** |
| **S (Ser)** | **5.5** (1.2) | **6.2** (0.8) | **8.4** (0.7) | ***** +** | ***** +** | ***** +** |
| **pol** | **27.9** (2.2) | **31.0** (1.6) | **33.8** (1.7) | ***** +** | ***** +** | ***** +** |
| **pol-char** | **1.4** (3.8) | **6.4** (2.0) | **7.9** (1.9) | ***** +** | ***** +** | ***** +** |
| **Q (Gln)** | **2.3** (0.9) | **3.8** (0.8) | **4.2** (0.9) | ***** +** | ***** +** | **** +** |
| **T (Thr)** | **4.6** (0.5) | **5.4** (0.5) | **5.6** (0.5) | ***** +** | ***** +** | *** +** |
| **C (Cys)** | **0.8** (0.3) | **1.0** (0.3) | **1.7** (0.5) | *** +** | ***** +** | ***** +** |
| **D (Asp)** | **4.9** (0.6) | **5.4** (0.7) | **5.4** (0.5) | ***** +** | ***** +** | **ns** |
| **P (Pro)** | 4.6 (0.9) | **4.1** (0.9) | **5.2** (1.1) | *** -** | **** +** | ***** +** |
| **N (Asn)** | 3.6 (1.3) | 4.4 (1.6) | 4.9 (2.2) | *** +** | **** +** | **ns** |
| **char** | **26.5** (2.0) | **24.6** (1.3) | **25.9** (1.1) | ***** -** | **ns** | ***** +** |
| **R (Arg)** | 5.9 (1.5) | 5.0 (1.5) | 5.4 (1.2) | *** -** | *** -** | **ns** |
| **M (Met)** | 2.3 (0.4) | 2.4 (0.4) | 2.2 (0.2) | **ns** | **ns** | *** -** |
| **K (Lys)** | 6.4 (2.3) | 5.8 (2.4) | 6.1 (1.6) | **ns** | **ns** | **ns** |
| **F (Phe)** | 4.2 (0.7) | 4.3 (0.8) | 4.0 (0.5) | **ns** | **ns** | **ns** |
| **W (Trp)** | 1.1 (0.3) | 1.1 (0.3) | 1.2 (0.3) | **ns** | **ns** | **ns** |
