# Supplementary figures and images for: Evolution of proteomes: fundamental signatures and global trends in amino acid compositions
Source: BMC Genomics. 2006 Dec 5;7:307. doi: 10.1186/1471-2164-7-307 (PMC1764020; doi:10.1186/1471-2164-7-307)

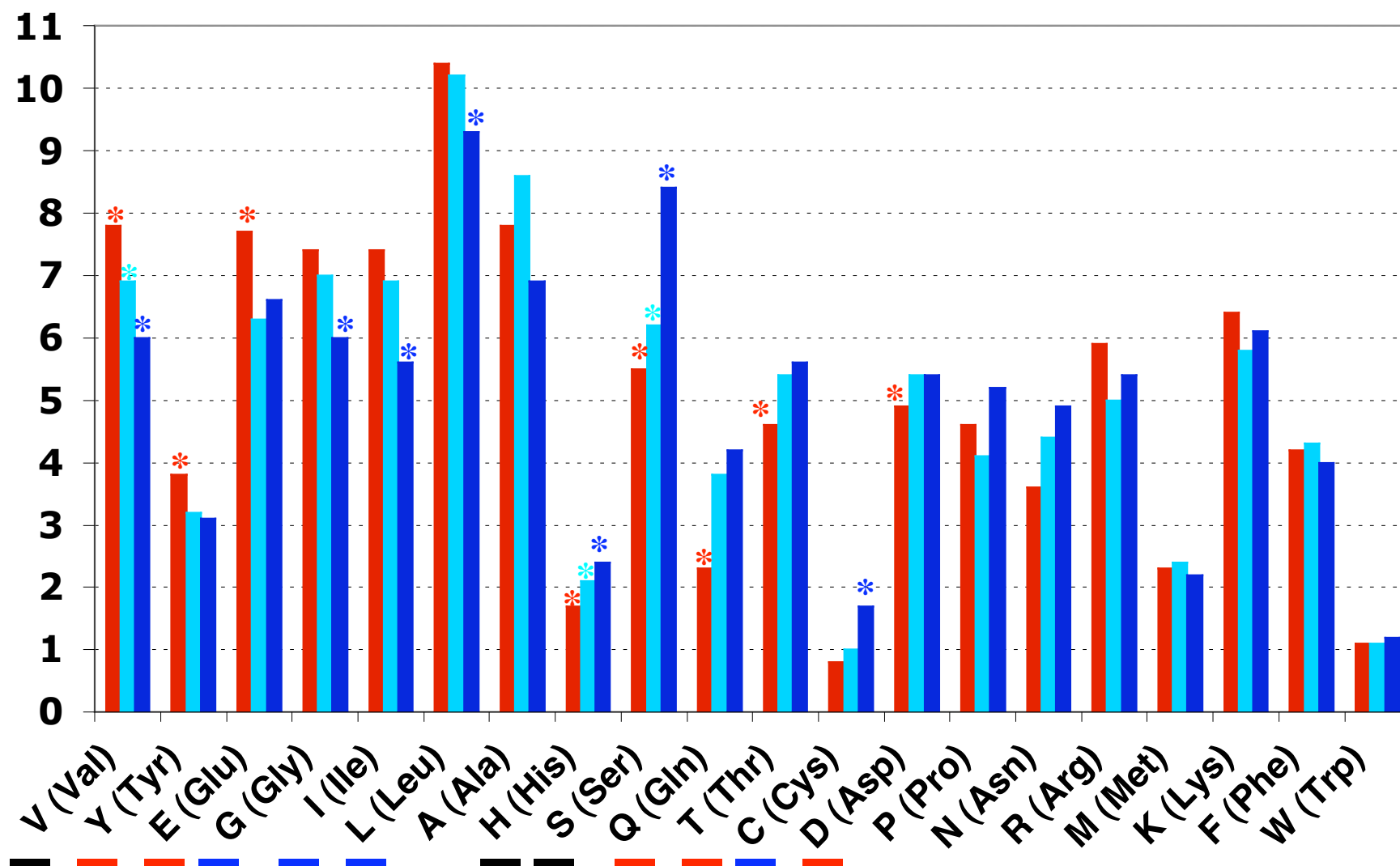

Supplement: Additional file 3 — Mean compositions for each amino acid in each of the three groups: Hyperthermophiles-thermophiles (red), Prokaryotic mesophiles-psychrophiles (light blue) and Eukaryotes (blue). Mean values for each amino acid in each of the three groups: Hyperthermophiles-thermophiles (red), Prokaryotic mesophiles-psychrophiles (light blue) and Eukaryotes (blue). Symbol * is associated with significant Newman-Keuls multiple comparison tests at the probability p < 0.001 (see Methods). Amino acids underlined with black, red and blue are characteristic respectively, of the three groups (Hyperthermophiles-thermophiles; Prokaryotic mesophiles-psychrophiles and Eukaryotes), of Hyperthermophiles only and of Eukaryotes only. [file 1471-2164-7-307-S3.pdf]

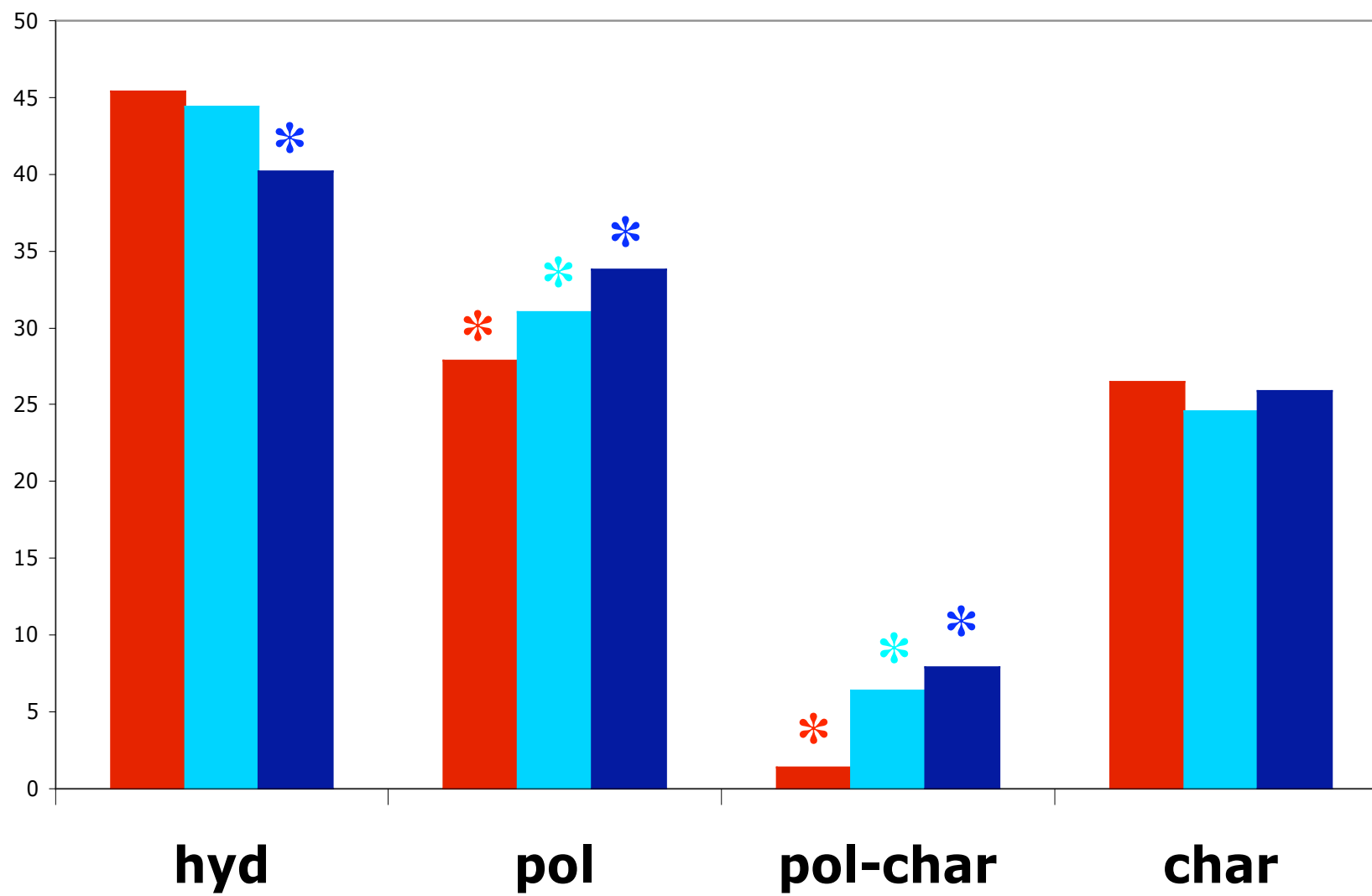

Supplement: Additional file 4 — Mean amino acid compositions according to physico-chemical properties. This figure shows mean values for hyd (hydrophobics), pol (polar), pol-char (pol – char) and char (charged) amino acids in each of the three groups: Hyperthermophiles-thermophiles; Prokaryotic mesophiles-psychrophiles and Eukaryotes. Colours are as in Additional file 3. Symbol * is associated with significant Newman-Keuls multiple comparison tests at p < 0.001 (see Methods). pol and [pol-char] are specific for each of the three groups. [file 1471-2164-7-307-S4.pdf]
